# *Camellia sinensis* (L.) Kuntze Extract Ameliorates Chronic Ethanol-Induced Hepatotoxicity in Albino Rats

**DOI:** 10.1155/2014/787153

**Published:** 2014-08-31

**Authors:** Poonam Lodhi, Neeraj Tandan, Neera Singh, Divyansh Kumar, Monu Kumar

**Affiliations:** ^1^Environmental Endocrinology and Biomedical Research Unit, Department of Zoology, Meerut College, Meerut 250003, India; ^2^Scientific and Applied Research Center, Meerut 250001, India; ^3^D.A.V. (P.G.) College, Bulandshahr 203001, India

## Abstract

The goal of this study was to investigate the hepatoprotective effects of aqueous extract of *Camellia sinensis* or green tea extract (AQGTE) in chronic ethanol-induced albino rats. All animals were divided into 4 groups in the study for a 5-week duration. 50% ethanol was given orally to the rats with two doses (5 mg/kg bw and 10 mg/kg bw) of AQGTE. Ethanol administration caused a significant increase in the levels of plasma and serum enzymatic markers, alanine aminotransferase (ALT), aspartate aminotransferase (AST), and alkaline phosphatase (ALP), and nonenzymatic markers (cholesterol and triglycerides), lipid peroxidation contents, malondialdehyde (MDA), and glutathione-S-transferase (GST), and decreased the activities of total proteins, albumin, and cellular antioxidant defense enzymes such as superoxide dismutase (SOD). The elevation and reduction in these biochemical enzymes caused the damage in hepatocytes histologically due to the high production of ROS, which retards the antioxidant defense capacity of cell. AQGTE was capable of recovering the level of these markers and the damaged hepatocytes to their normal structures. These results support the suggestion that AQGTE was able to enhance hepatoprotective and antioxidant effects *in vivo* against ethanol-induced toxicity.

## 1. Introduction

Alcohol is widely consumed in alcoholic drinks in modern society, and ethanol is one of the main causes of a variety of medical problems and liver diseases worldwide [1]. The liver is the major target organ of ethanol toxicity [2]. Chronic ethanol feeding causes a decrease in the major antioxidant factors in the liver, including enzymes [3, 4] and nonenzymatic antioxidants [5, 6]. This is due to the generation of an excessive amount of reactive oxygen species (ROS), which results in the detrimental effects of the cellular antioxidant defense system [7, 8]. Thus, excess alcohol consumption may accelerate an oxidative mechanism directly or indirectly, which eventually produces cell death and tissue damage [9–13]. Therefore, alternative treatments for liver disorders are needed to replace the existing synthetic drugs.

The plants having antioxidants prevent the cell death and tissue damage resulting from chronic alcohol consumption [14].

Green tea (*Camellia sinensis*, Theaceae) is the second most popular beverage worldwide [15]. It contains six primary catechins or polyphenol compounds. These constituents have potent antioxidant action and their putative disease preventive effects [16]. These polyphenols prevent oxygen-free radicals-induced hepatocyte lethality, reduce the risk of liver disease, and protect against liver injury, that is, fibrosis and liver cirrhosis in rats [17–19]. In the current study, we evaluated the influence of green tea extract on liver specific enzymatic and nonenzymatic markers, lipid peroxidation, antioxidants in blood, and liver histology associated with chronic ethanol consumption.

## 2. Materials and Methods

### 2.1. Chemicals

Ethanol (purity (GC) ≥ 99.9%) and all other chemicals were from Merck (Merck KGaA, Darmstadt, Germany). All chemicals used were of analytical grade.

### 2.2. Animal Treatment

Twenty-four healthy male Wistar strain rats (100–140 g; 14–16 weeks old) were obtained from animal house of C.C.S, University, Meerut, U.P., India. All animals were acclimatized for laboratory conditions at room temperature and kept on normal diet. The tap water ad libitum was also provided for two weeks before starting the experiment. All animals were cared for according to guidelines of the Institutional Animal Ethics registered by IAEC (384/PO/a/01/CPCSEA 27-03-2015). Committee (IAEC) and experiments were also approved. The experiment duration was 5 weeks. All animals were divided into 4 groups: controls (received distilled water as drinking source), ethanol control (0.5 mL C_2_H_5_OH/100 gm body weight), ethanol + GTE (0.5 mL C_2_H_5_OH + 5 mg GTE/100 gm body weight), and ethanol + GTE (0.5 mL C2H5OH + 10 mg GTE/100 gm body weight). The animals of alcohol control group were administered oral dose of ethanol everyday between 10:00 AM and 11:00 AM. Experimental animals of group 3 and group 4 were given orally 5 mg and 10 mg/100 gm body weight dose of GTE, respectively, after 1 hour of feeding of alcohol.

*Camellia sinensis* was procured from Tea State of Tata Group of Company, TALAT, Assam, H.P. (India). Preparation of GTE was done according to the method described by [20]. Green tea is prepared by picking the leaves, lightly steaming them, and allowing them to dry. The dried material was ground into powder using mortar and pestle and sieved with a sieve. 100 g of the powdered plant material was steeped in 600 mL of distilled water and heated in water bath for 3 h at 90°C. The mixture was allowed to cool to room temperature, filtered, and dried. Total 3 gm of yield of dried GTE was obtained. This GTE was used to treat rats.

At the end of study, body weights of all animals were recorded, then sacrificed under light ether anesthesia, and dissected. The blood samples were collected by retroorbital plexus in fluoride and plain glass tubes. Liver was removed, weighed, and processed for biochemical studies. Blood samples from rats were immediately centrifuged at 3000 rpm for 10 min at 4°C for serum samples. The supernatant of serum was separated from the pellet and used for biochemical analysis.

### 2.3. Histopathological Examination

After scarification of rats, the samples of liver tissues were collected from all groups. For histological study, they were fixed in 10% neutral buffered formalin, dehydrated in ascending grades of ethanol alcohols, cleared in xylol, casting, blocking, cutting at 5 *μ*m thickness, and stained [21]. For the homogenate, liver was removed quickly and kept in iced 0.15 M NaCl solution, for removing the blood cells, blotted on filter paper, weighed, and homogenized. The supernatant was kept on ice until assayed after centrifugation of homogenates at 10,000 g for 15 min at 4°C.

### 2.4. Biochemical Analysis

#### 2.4.1. Measurement of Blood Alcohol

Blood was taken from the tail vein 1 h after gavage, 2 weeks after initiation of alcohol. Blood alcohol levels (BAL) were measured using the alcohol dehydrogenase kit from Sigma Chemical Co., India [22].

#### 2.4.2. Determination of Liver Enzymatic Markers

The levels of liver enzymes were measured using commercial kits (Sigma Chemical Co., India). The plasma levels of alanine transaminase (ALT) and aspartate transaminase (AST) were estimated according to [23] and alkaline phosphatase (ALP) was according to [24].

#### 2.4.3. Determination of Liver Nonenzymatic Markers

The levels of cholesterol and triglycerides were measured by using standard assay kits (Sigma Chemical Co., India).

#### 2.4.4. Determination of Total Proteins Levels

Estimation of total proteins levels in plasma and in liver tissue homogenates was measured by the method previously described using bovine serum albumin as the standard [25]. The plasma levels of albumin were determined using commercial kit (Sigma Chemical Co., India) according to Pinnell and Northam colorimetric method [26].

#### 2.4.5. Determination of Liver MDA Contents

The tissues of liver were thawed, weighed, and homogenized 1 : 9 w : v in 0.9% saline. Then the homogenates were centrifuged at 3000 rpm for 10 min at 4°C in a high-speed centrifuge and the supernatant was taken for the assays of MDA (a measure of lipid peroxidation) contents. MDA was assayed by the measurement of thiobarbituric acid-reactive substances (TBARS) levels spectrophotometrically at 532 nm. Results were expressed as nmol·mg^−1^ protein [27].

#### 2.4.6. Determination of Antioxidants

The level of superoxide dismutase (SOD) in liver tissue homogenate was assayed according to [28]. Level of GST in liver homogenates was measured according to Habig et al. [29].

### 2.5. Statistical Analysis

The results were presented as mean ± SD. Statistical significance and differences from control and test values were evaluated by Student's *t*-test. Statistical probability *P* < 0.05 was considered as statistically significant. Statistical analysis was conducted by using Sigma Plot software (Version 11).

## 3. Results

### 3.1. Measurement of Blood Alcohol

The rats increased their weight at a constant rate in each of the groups studied; there was no difference in weight gain among the groups. At week 2, BAL 1 h after ethanol administration by gavage was similar in alcohol/GTE group (376.6 ± 68.1 mg/100 mL) and alcohol group (387.3 ± 51.9 mg/100 mL).

### 3.2. GTE Effect on C_2_H_5_OH Induced Changes in Liver Enzymatic Markers

The levels of ALT, AST, and ALP were evaluated in albino rats serum. As shown in Tables 1 and 2, a single dose of EtOH (0.5 mL/100 gm body weight) caused hepatotoxicity. The levels of these enzymatic markers in ethanol control group were increased significantly as compared to control groups. These increased levels were due to the liver cell injuries induced by EtOH. Administration of GTE significantly prevented the EtOH-induced elevation of serum ALT, AST, and ALP levels.

### 3.3. GTE Effect on C_2_H_5_OH Induced Changes in Liver Nonenzymatic Markers

The cholesterol and triglycerides level of untreated ethanol control group was significantly higher than the other experimental groups (Table 3). In contrast, the levels of cholesterol and triglycerides of the 5 mg/kg body weight of GTE with EtOH groups were significantly lower than the ethanol control group. Added to this, 10 mg/kg body weight of GTE with EtOH was able to reduce the level of these nonenzymatic markers to near the normal value, whereas the level of urea was normal till the duration of experiments.

### 3.4. Determination of Total Proteins Levels in Liver

The level of total proteins depends upon the addition of albumin and globulin levels. The level of albumin in ethanol group was significantly lower than the control group, whereas the globulin level was normal. The level of albumin in 5 mg/kg body weight of GTE with EtOH groups was significantly higher, whereas 10 mg/kg body weight dose of GTE was able to increase the level of albumin to near the normal value when compared with ethanol and control groups.

### 3.5. GTE Effect on C_2_H_5_OH Induced Changes in Liver MDA Contents

The level of MDA (Table 4) in ethanol control group was significantly increased as compared to control groups. The increased level of MDA in ethanol control group indicated the presence of lipid peroxidation of liver cells, which was due to the toxic effect of EtOH. Furthermore, all of the GTE treatment groups were significantly different as compared to ethanol control group, and the MDA content was normalized as compared to normal control and ethanol control groups. Both doses of GTE were able to reduce the level of MDA as compared to normal control.

### 3.6. GTE Effect on C_2_H_5_OH Induced Changes in Activity of Antioxidant Enzymes

Table 4 summarizes the activities of hepatic antioxidant enzymes. Chronic EtOH administration to rats caused a significant decrease in the activities of SOD and a significant increase in the level of GST. 5 mg doses of GTE were able to increase the level of SOD and decrease the level of GST as compared to ethanol control. 10 mg doses of GTE were able to normalize the levels of SOD and GST as compared to control groups and showed a spectacular restoration of hepatic SOD and GST activities.

### 3.7. Histopathological Examination

**Figure 1:**
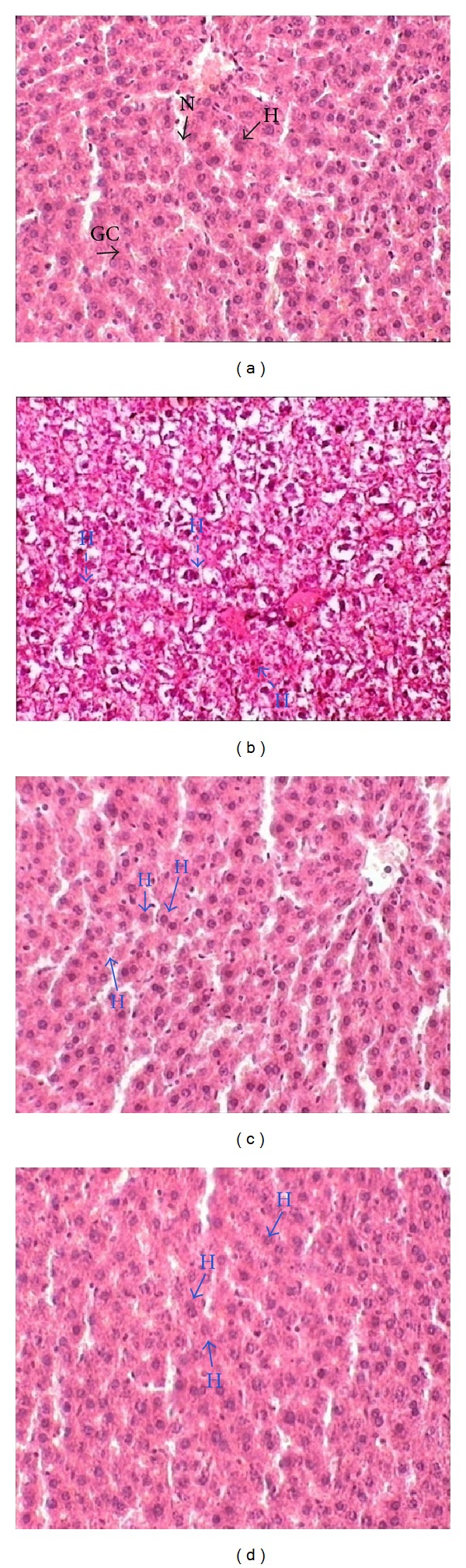
Microsection of liver of different experimental groups showing normal structure having hepatocytes (H) with granular cytoplasm (GC), and clear nucleus (N) was stained with hematoxylin and eosin with the magnification of 400x. (a) Normal control with normal structure of liver cells. (b) Ethanol control with several microvacuolations of hepatocytes condition as indicated by the dashed line arrows. (c) 0.5 mL EtOH + 5 mg/100 gm AQGTE-treated group with recovery effect from microvacuolation of hepatocytes. (d) 0.5 mL EtOH + 10 mg/100 gm AQGTE-treated group with recovery effect from microvacuolation of hepatocytes. H = hepatocyte, S = sinusoidal space, hepatocyte developed with microvacuolation.

The liver sections from different experimental groups were used to observe histopathological changes which were stained with H and E staining and observed via a light microscope (Figure 1). In normal control group (Figure 1(a)), normal hepatocytes were observed, while, in ethanol control (Figure 1(b)), the hepatocytes showed fatty changes which were also known as microvesicular steatosis where the hepatocytes developed an open space around the nuclei. The administration of 5 mg/kg and 10 mg/kg body weight of GTE with EtOH showed a recovery effect of hepatocytes with minimal to no microvesicular steatosis. These results showed that GTE treatment resulted in a protective effect on EtOH-induced hepatotoxicity.

## 4. Discussion

Ethanol is a hepatotoxicant to induce liver damage since it is clinically relevant [30]. This liver damage is associated with several reactions of free radicals such as reactive oxygen species (ROS) which causes elevation in MDA and GST content while reducing the level of SOD. The elevation and reduction of enzymatic and non-enzymatic markers of serum were also associated with this condition. Alcoholic liver disease was normally found in liver histology. GTE was enriched with antioxidants that could revert and lower the free radicals level. It had shown that the beneficial effects of this phytochemical in preventing the ethanol-induced hepatotoxicity are mediated by the antioxidant effects.

In the present study, we evaluated the protective effects of GTE against ethanol-induced hepatotoxicity. Ethanol administration increased the BAL, which caused the changes in the behavior of rats. Both doses of green tea extract normalized this change in BALs [22]. Ethanol administration elevated the concentrations of key cellular enzymes like AST, ALT, and ALP present in the liver cells leak into the serum during liver damage [31–35]. Elevated activities of these enzymes indicate hepatocytes damage where the leakage of cell membrane participated in the accumulation of these enzymes into the plasma [36, 37]. This is because of higher concentration of alcohol dehydrogenase in liver, which catalyzes alcohol to its corresponding aldehyde [38]. GTE had the ability to reduce the level of these enzyme markers. Therefore, prolonged treatment of 10 mg/100 gm body weight of GTE administration could help to normalize the ALT, AST, and ALP enzyme levels. Our results are also consistent with protective effects of different extracts with antioxidant ability against alcohol-induced hepatocyte cells of liver [20, 35, 39–41]. Ethanol administration decreased (serum protein and albumin) and increased (cholesterol and TG) the levels of nonenzymatic markers which caused the liver damage. This damage is attributed to the higher concentration of alcohol dehydrogenase enzyme which catalyses alcohol to aldehyde and accumulation of export type proteins due to inhibition of the secretion of the proteins from the liver of alcoholics [38, 42]. Both doses of GTE restored the low level of protein in a dose dependent manner to normal level [43–45]. Level of cholesterol was increased with ethanol and decreased with both doses of GTE in all treatment groups [46].

Liver histology of such experimental animals also showed improvement (Figure 1). In this study these biochemical tests were supported by histopathological observations of liver sections [47].

The level of MDA and GST in cirrhotic rats was found to be high as compared to the controls [48, 49]. The levels of MDA and GST content were low in GTE treated group (Table 4) as polyphenol rich green tea extracts inhibit lipid peroxidation in experimental rats [50]. During this study, the antioxidant system of cirrhotic rats was severely impaired, causing a high level of MDA and GST. The oxidative tissue damage in cirrhosis causes a significantly low level of catalase. During the process of inflammation, oxidative stress occurs which leads to a significant decrease in antioxidant enzyme system. The main target of oxidative stress is the poly unsaturated fatty acids in cell membranes causing lipid peroxidation and excessive formation of MDA and GST which may lead to damage of the cell structure and function [51]. The low level of SOD indicates that the high risks of cell injuries and the treatment of GTE doses tend to increase or normalize the level of SOD. The level of SOD in the liver of alcohol-induced mice treated with both doses of GTE was observed to be increased. In addition, this signified that GTE could provide the elevated SOD enzyme to the injured liver cells which in turn could recover the liver cells to normal and eventually be able to produce the significant amount of SOD enzyme by itself as a protective action from the damage caused by toxic substance such as alcohol [47]. Chronic alcohol consumption not only activates free radical generation, but also alters the levels of both enzymatic and nonenzymatic endogenous antioxidant systems [14].

## 5. Conclusion

In conclusion, in the present study, we investigated that the natural antioxidants present in AQGTE ameliorate liver damage caused by chronic ethanol exposure. These results were more effective in reverting the enzymatic markers (AST, ALT, and ALP), nonenzymatic markers in liver homogenate (protein, cholesterol, and triglycerides), antioxidant activity in liver homogenate (MDA, GST, and SOD), and histological conditions back to normal. It shows that GTE has the capability to prevent this toxicity by inhibiting the hepatocyte damage, peroxidation of lipids, and improving the activity of antioxidant enzymes.

## Figures and Tables

**Table 1:** Effect of AQGTE on concentrations of nonenzymatic biochemical parameters in blood in chronic ethanol-induced hepatic damage in rats.

Analyzed parameters	Controls	EtOH group	EtOH + GTE group	EtOH + GTE group
		**0.5 mL/kg B wt.**	**0.5 mL + 5 mg/kg B wt.**	**0.5 mL + 10 mg/kg B wt.**
AST (IU/L)	22.00 ± 3.139	46.50 ± 2.715***	42.50 ± 2.184****	28.16 ± 2.119***
ALT (IU/L)	39.00 ± 2.678	100.00 ± 5.319***	54.83 ± 4.148***	24.83 ± 1.678***
ALP (KA Units/100 mL)	104.00 ± 8.871	144.00 ± 3.551***	130.50 ± 3.303***	105.83 ± 4.548***

Values represent mean ± SEM; *n* = 6; significance as per Student's *t*-test.

*P < 0.01, **P < 0.005, ***P < 0.001, ∗∗∗∗ nonsignificant, ∗∗∗∗∗ no change.

**Table 2:** Effect of AQGTE on concentrations of nonenzymatic biochemical parameters in liver tissue in chronic ethanol-induced hepatic damage in rats.

Analyzed parameters	Controls	EtOH group	EtOH + GTE group	EtOH + GTE group
		**0.5 mL/kg B wt.**	**0.5 mL + 5 mg/kg B wt.**	**0.5 mL + 10 mg/kg B wt.**
AST (IU/L)	1458 ± 119.5	2564 ± 116.42***	24150 ± 111.20****	2398 ± 87.29***
ALT (IU/L)	2230 ± 146.1	2564 ± 116.42***	2415 ± 111.45***	2398 ± 87.89***
ALP (KA Units/100 mL)	108.00 ± 8.867	136.16 ± 1.490***	129.66 ± 0.98	124.01 ± 1.06∗∗∗

Values represent mean ± SEM; *n* = 6; significance as per Student's *t*-test.

*P < 0.01, **P < 0.005, ***P < 0.001, ∗∗∗∗ nonsignificant, ∗∗∗∗∗ no change.

**Table 3:** Effect of AQGTE on concentrations of nonenzymatic biochemical parameters in liver tissue in chronic ethanol-induced hepatic damage in rats.

Analyzed parameters	Controls	EtOH group	EtOH + GTE group	EtOH + GTE group
		**0.5 mL/kg B wt.**	**0.5 mL + 5 mg/kg B wt.**	**0.5 mL + 10 mg/kg B wt.**
Cholesterol (mg/dL)	56.21 ± 5.279	76.54 ± 2.364*	67.65 ± 1.89****	57.30 ± 1.115***
Triglycerides (U/L)	4.70 ± 0.218	32.50 ± 1.453***	21.26 ± 0.459****	6.03 ± 0.490***
Total protein (g/dL)	4.06 ± 0.816	2.52 ± 0.159***	3.55 ± 0.216****	3.91 ± 0.131***
Albumin g/dL	2.7 ± 0.196	1.38 ± 0.106***	1.88 ± 0.217****	2.07 ± 0.216***
Globulin g/dL	1.38 ± 0.186	1.12 ± 0.077****	1.68 ± 0.245****	1.86 ± 0.216****
Urea (mg/dL)	22.01 ± 1.261	23.00 ± 0.633*****	24.00 ± 1.63	26.33 ± 2.85

Values represent mean ± SEM; *n* = 6; significance as per Student's *t*-test.

*P < 0.01, **P < 0.005, ***P < 0.001, ∗∗∗∗ nonsignificant, ∗∗∗∗∗ no change.

**Table 4:** Effect of AQGTE on concentrations of MDA content and activity of antioxidant enzymes in liver tissue in chronic ethanol-induced hepatic damage in rats.

Analyzed parameters	Controls	EtOH group	EtOH + GTE group	EtOH + GTE group
		**0.5 mL/kg B wt.**	**0.5 mL + 5 mg/kg B wt.**	**0.5 mL + 10 mg/kg B wt.**
MDA (nmol/mg protein)^1^	4.20 ± 0.068	10.20 ± 0.483***	6.00 ± 0.266****	3.90 ± 0.085***
SOD (U/mg protein)^1^	16.15 ± 0.054	11.44 ± 0.168***	14.54 ± 0.145****	18.36 ± 0.131***
GST (*μ*g/min/mg protein)	3.23 ± 0.154	6.96 ± 0.205***	4.20 ± 0.169****	3.46 ± 0.023***

Values represent mean ± SEM; *n* = 6; significance as per Student's *t*-test.

*P < 0.01, **P < 0.005, ***P < 0.001, ∗∗∗∗ nonsignificant, ∗∗∗∗∗ no change.

^1^Number of nmol per 1.
